# Once a clinician, always a clinician: a systematic review to develop a typology of clinician-researcher dual-role experiences in health research with patient-participants

**DOI:** 10.1186/s12874-016-0203-6

**Published:** 2016-08-09

**Authors:** E. Jean C. Hay-Smith, Melanie Brown, Lynley Anderson, Gareth J. Treharne

**Affiliations:** 1 Department of Medicine, University of Otago, Wellington, PO Box 7343, Wellington, 6242 New Zealand; 2 Bioethics Centre, University of Otago, Otago, New Zealand; 3 Department of Psychology, University of Otago, Otago, New Zealand

**Keywords:** Clinician-researcher, Dual role, Reflexivity, Research ethics, Systematic review, Meta-synthesis

## Abstract

**Background:**

Many health researchers are clinicians. Dual-role experiences are common for clinician-researchers in research involving patient-participants, even if not their own patients. To extend the existing body of literature on why dual-role is experienced, we aimed to develop a typology of common catalysts for dual-role experiences to help clinician-researchers plan and implement methodologically and ethically sound research.

**Methods:**

Systematic searching of Medline, CINAHL, PsycINFO, Embase and Scopus (inception to 28.07.2014) for primary studies or first-person reflexive reports of clinician-researchers’ dual-role experiences, supplemented by reference list checking and Google Scholar scoping searches. Included articles were loaded in NVivo for analysis. The coding was focused on how dual-role was evidenced for the clinician-researchers in research involving patients. Procedures were completed by one researcher (MB) and independently cross-checked by another (JHS). All authors contributed to extensive discussions to resolve all disagreements about initial coding and verify the final themes.

**Results:**

Database searching located 7135 records, resulting in 29 included studies, with the addition of 7 studies through reference checks and scoping searches. Two overarching themes described the most common catalysts for dual-role experiences – ways a research role can involve patterns of behaviour typical of a clinical role, and the developing connection that starts to resemble a clinician-patient relationship. Five subthemes encapsulated the clinical patterns commonly repeated in research settings (clinical queries, perceived agenda, helping hands, uninvited clinical expert, and research or therapy) and five subthemes described concerns about the researcher-participant relationship (clinical assumptions, suspicion and holding back, revelations, over-identification, and manipulation). Clinician-researchers use their clinical skills in health research in ways that set up a relationship resembling that of clinician-patient. Clinicians’ ingrained orientation to patients’ needs can be in tension with their research role, and can set up ethical and methodological challenges.

**Conclusion:**

The typology we developed outlines the common ways dual-role is experienced in research involving clinician-researchers and patient-participants, and perhaps the inevitability of the experience given the primacy accorded to patient well-being. The typology offers clinician-researchers a framework for grappling with the ethical and methodological implications of dual-role throughout the research process, including planning, implementation, monitoring and reporting.

## Background

Health research frequently addresses questions derived from clinical practice. When clinicians are involved in research there are a number of benefits including: increased clinical relevance of research questions, gaining access to clinical settings for research, bringing clinical expertise and insider perspectives to the research, having researchers who are trusted by participants which may encourage their participation, and having researchers who are motivated to disseminate applicable findings and continue their commitment to the researched [1].

A clinician-researcher is an individual who conducts research and provides direct patient care [2], although not necessarily at the same time or for the same organisation. Regardless of whether or not there is a pre-existing clinical relationship between the clinician-researcher and the patient-participant, the ethical and methodological implications of clinicians undertaking research are particularly challenging when conducting studies that involve direct patient contact [3]. Expectations, orientations and competing obligations mean that clinician-researchers are likely to experience situations in which their sense of clinical duty comes into apparent tension with research ethics or methodological demands, and this triggers dual-role experiences such as role confusion [4]. Role confusion can be both external (clarifying roles to others) and internal (feeling conflict between roles) [2].

A clinician-patient relationship contains a number of features that can give rise to problems when suddenly transformed from a clinical relationship to a research relationship, both real and perceived [5]. Patient-participants bring the experience and memory of a patient-clinician relationship to the research setting [6]. The clinician-researcher has ingrained values, skills and knowledge derived from intensive professional socialization that makes it difficult to wholly divorce from the care and welfare of patients [4].

Clinician-researchers are bound by the ethical norms of both clinical practice and research [7]. The dual nature of the clinician-researcher role means that in addition to advantages that the transferability of clinical skills and attributes bring to the research setting, there is need to ensure the clinician-researcher’s privileged position is balanced with responsibility both to patient-participants and rigorous research methods [8]. There are differences and similarities between ethical requirements of doing research and providing treatment [9]. Nevertheless, health professional codes of ethics (e.g. General Medical Council [10]) and principles of ethical medical research with human participants [11] mean that clinicians have a duty to act in accordance with patient wishes and best interests, and put patient well-being first in research.

Empirical studies and scholarly reviews on dual-role in clinician-researchers have largely focused on why dual-role occurs (such as tension between ethical frameworks) and ways to prevent or manage it (such as careful consideration of recruitment and informed consent processes in research design). We were unable to find any published descriptive classification of the most common ways dual-role is experienced by clinician-researchers in clinical research with patient-participants. We did find reports of dual-role arising in qualitative and in quantitative research designs. Thus, the aim of our review was to locate and synthesise existing reports of dual-role experiences arising in qualitative and quantitative research to develop a typology of the typical manifestations of dual-role for clinicians undertaking research involving patients.

## Methods

Electronic databases (Medline, CINAHL, PsycINFO, Embase, and Scopus) were searched (date of inception until 28 July 2014) for primary studies (i.e. reporting data collected about dual-role experiences) or first-person accounts (i.e. author reflecting on their own research experience of dual-role) of clinician-researcher experiences of dual-role. Developing the search strategy proved difficult, with highly variable text and keywords and subject heading, across papers, disciplines and databases. The search strategy was developed iteratively in consultation with a medical librarian, starting with Google Scholar scoping searches later tailored to each database. Database-specific subject headings, text and keywords covered three topics: researcher-clinician; participant-patient; dual-role relationship. Examples of the latter included variations of role conflict, double agent, dual or multiple role or relationship, and blurred boundaries. Search strategies are available in Additional file 1. Database searching was supplemented by reference list checking and further Google Scholar scoping searches because the difficulties balancing search specificity and sensitivity meant that eligible papers might be missed.

Inclusion criteria were: English language (due to the barrier of translation costs); health research in patient settings; primary studies and first-person accounts detailing examples of how dual-role was experienced by clinician-researchers. We define clinician as a member of a registered health profession involved in direct patient care (e.g. medical doctor, nurse, allied health professional). We excluded scholarly papers without first-person data about clinician-researcher experiences of dual-role; papers where researchers were not clinicians (or it was not clear); research including clinician-researchers and non-clinician-researchers where the experiences of the two groups could not be differentiated; and health-related research in non-patient settings (e.g. outreach with sex workers).

Search results were imported into Endnote, and after de-duplication all records were screened for eligibility by one researcher (MB). Another (JHS) independently checked a random sample (12% of total) to test the screening tool before screening was completed. Disagreements about eligibility were resolved through discussion and decision rules recorded to support subsequent judgements.

Reports were loaded into NVivo-10 and all instances of dual-role experience coded. The coding focus was to ask what was happening (researcher or participant behaviour, researcher feelings or thoughts) when dual-role was experienced by the clinician-researcher. An iterative approach to thematic analysis was used [12]; initial candidate themes were informed by the researchers’ previous inductive thematic analysis of the literature in an earlier unpublished scoping review on the nature of dual-role (Hay-Smith EJC, Personal Communication) and iteratively adapted and added to by themes inductively derived from the coded data.

Specifically, the analytic stages were: (1) coding completed by one researcher (MB) and independently crosschecked by another (JHS), with all disagreements resolved through discussion. Initial codes were generated and discussed by the two researchers (MB, JHS) using deductive and inductive techniques; (2) data in each coding category were examined and higher level codes developed (and data re-coded if necessary) through discussion in four regularly spaced meetings of all researchers; (3) codes were grouped into themes with a central organizing concept and agreed descriptor, and each article was repeatedly re-examined for instances of the emerging themes; and (4) candidate themes were reviewed in relation to the whole dataset and data comparison was used to check for relationships between themes. Ten themes (and two overarching themes) were iteratively developed and agreed by all four researchers. Representative quotes for each theme were also agreed. For purposes of brevity, and where it seemed not to alter meaning, we occasionally omitted a short section of a longer quote (denoted by three en dashes, − − –).

The first-person accounts were not appraised for ‘quality’ as we likened these reports to the type of data gathered in an interview – that is, the clinician-researcher’s interpretation of their own experience. On the other hand, primary studies presented the researchers’ interpretation of the clinician-researchers’ experiences and accordingly were influenced by the researchers’ assumptions, methodology, and research purpose. Primary studies reporting thematic or content analyses were critically appraised by one author (JHS) and cross-checked by another (GT), using items 3–9 of the Critical Appraisal Skills Programme (CASP) Qualitative Research Checklist (version 31.05.13) [13]. We (JHS, GT) agreed on the credibility, dependability, confirmability, and transferability of each report (graded low, moderate, high for each component), and then categorised each primary study overall as trustworthy, reasonably so, or uncertain. In confirming each theme we noted what proportion of papers and data originated from primary reports (and the overall quality rating) and whether the data from the primary studies were congruent with that from first person accounts.

## Results

**Fig. 1:**
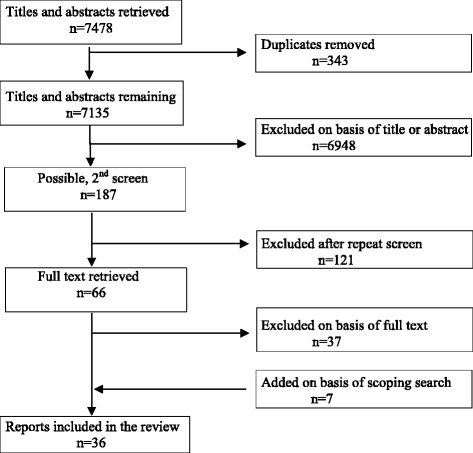
Search and screening results

After de-duplication, 7135 search records were screened for eligibility based on title only (*n* = 587) or title and abstract (*n* = 6548) (Fig. 1). Sixty-six full papers were retrieved, of which 29 were included. Seven of 10 further papers identified from reference lists and scoping searches were included. Another article was a second publication containing complementary data [7] from an already included study [14]; we extracted and coded data from this but did not count it as a ‘separate’ report. The two remaining articles were excluded; one [15] because it was a companion publication (on ethics) to an already included study [16], and the other [17] was an audit of requests for research protocol exceptions arising from dual-role conflict but contained no data on how dual-role was experienced.

Twenty eight reports were first-person accounts and 8 were primary studies of clinician-researcher experiences of dual-role (Table 1). Nursing research dominated, as 20 of the 28 first person accounts were from nurse-researchers and 7 of the 8 primary studies recruited nurse-researchers. Most (*n* = 26) of the first-person accounts were reflections on dual-role experienced in qualitative research, and 2 concerned experiences in randomised trials. The 8 primary studies of dual-role all used qualitative methods; one was categorised as trustworthy, three as reasonably so, and four as uncertain (Table 1). The first-person accounts provided most of the detailed and ‘concrete’ examples of how dual-role was evident to clinician-researchers, and contributed the majority of coded data. Coded data are available in Additional file 2. Every theme contained data from first-person accounts (which always contributed the majority of data), and nine of the 10 themes included data from one to six primary studies. In each theme, data from the primary studies and first-person accounts were congruent and we concluded that the trustworthiness of individual primary studies had no particular influence on the findings of our thematic analysis.

**Table 1:** Included studies and contribution to themes

Study	Clinical patterns					Connection					Study description	
	1-Clinical Queries	2-Agenda Meeting	3-Helping Hands	4-Research or Therapy	5-Uninvited Clinical Expert	6-Clinical Assumptions	7-Suspicion and Holding Back	8-Revelations	9-Overidentification	10-Manipulation	Author (first-person), or sample (empirical) and setting	Methodology
First-person accounts												
Arber 2006 [52]			•								Nurse, palliative care	Ethnography
Baarnheilm and Ekblad 2002 [53]		•		•	•	•	•	•	•		Physician, psychiatry	Qualitative
Bailey 2007 [54]			•		•						Nurse, emergency department	Ethnography
Bland 2002 [30]			•		•	•		•		•	Nurse, nursing homes	Ethnography
Bonner and Tolhurst 2002 [26]			•								Nurse, nephrology	Participant observation
Burns et al. 2012 [22]		•	•		•				•		Midwife, postnatal care	Participant observation
Burr 1996 [28]				•				•	•		Nurse, intensive care	Qualitative
Cartwright and Limandri 1997 [55]	•			•	•			•		•	Nurse, elderly care	Grounded Theory
Clancy 2007 [39]	•			•		•				•	Nurse, home oxygen	Phenomenology
Clinton et al. 1986 [37]			•		•			•	•		Nurse, fathering	Multiple time-series survey
Colbourne and Sque 2004 [3]	•			•	•		•		•		Nurse, cancer care	Qualitative case study
Conneeley 2002 [35]	•	•		•	•	•	•		•	•	Occupational therapist, brain injury rehabilitation	Phenomenology
Gardner 1996 [6]		•				•			•	•	Nurse, hospital acquired infection	Qualitative
Groenkjaer 2002 [25]									•	•	Nurse, hospital wards	Critical ethnography
Hamburg and Johansson 1999 [20]		•			•	•			•	•	GP, primary care	Grounded Theory
Houghton et al. 2010 [56]			•		•						Nurse, hospital wards	Multiple case study
Lykkeslet and Gjengedal 2007 [36]					•				•	•	Nurse, surgery	Ethnography
McNair et al. 2008 [1]									•	•	GP, primary care	Critical phenomenology
Mitchell 2011 [29]				•	•			•			Midwife, pregnancy	Qualitative
Newbury 2011 [32]			•	•	•	•	•		•	•	Nurse, palliative care	Grounded theory approach
Nicholl 2007 [57]	•		•		•	•		•			Nurse, mothering	Qualitative
Patterson 1994 [33]		•	•	•					•	•	Nurse, nursing homes	Participant observation
Profitt et al. 1993 [16]	•	•	•		•				•		Nurse, stroke rehabilitation	Randomised trial
Richards and Emslie 2000 [38]	•	•		•		•	•	•			GP, primary care	Qualitative
Shaughnessy et al. 2007 [58]					•			•			Nurse, stroke rehabilitation	Randomised trial
Sterling and Peterson 2005 [34]	•	•	•	•				•	•		Nurse, family systems	Ethnography
Thompson and Russo 2012 [45]				•			•	•	•	•	Clinical psychologist, health settings	Qualitative
Tuffrey-Wijne et al. 2008 [59]				•	•				•		Nurse, learning disability	Ethnography
Empirical reports												
Beale and Wilkes 2001 [23]^b^	•		•	•	•			•	•		Nurses, various clinical research	Descriptive qualitative
Boase et al. 2011 [31]^a^						•			•		Nurses, diabetes research	Framework approach
Boydell et al. 2012 [14]^b^ and Czoli et al. 2011 [7]^b^					•						Physicians, paediatric research	Qualitative
Easter et al. 2006 [18]^c^				•	•						Physicians, nurses and patient-participants in gene transfer research	Qualitative
Haigh et al. 2005 [19]^c^	•	•		•		•					Nurse, post-surgery patient-participants	Case study
Johnson and Macleod Clarke 2003 [21]^c^	•	•		•	•			•		•	Nurses, cancer, HIV/AIDS, dying	Qualitative
Spilsbury et al. 2007 [60]^b^			•		•					•	Nurses, pressure mattress overlay research	Qualitative focus group
Wilkes and Beale 2005 [24]^c^			•	•							Nurses, various clinical research	Qualitative

Key: ^a^trustworthy, ^b^moderately trustworthy, ^c^trustworthiness uncertain, GP = general practitioner

Two overarching catalysts for dual-role experiences were identified. We called these ‘Clinical Patterns’ and ‘Connection’. Clinical Patterns describes instances of dual-role provoked when the clinician-researcher is asked, or chooses, to act as a clinical resource in the research setting for the benefit of the patient-participant. Acting as a clinical resource includes using clinical skills, clinical reasoning, and suggesting or referring a person to an information or care source. These clinical patterns are expressed in five themes: 1) Clinical Queries; 2) Perceived Agenda; 3) Helping Hands; 4) Uninvited Clinical Expert; 5) Research or Therapy.

Connection is comprised of five themes: 1) Clinical Assumptions; 2) Suspicion and Holding Back; 3) Revelations; 4) Over-Identification; 5) Manipulation. The common element in these themes is that while the primary relationship is researcher-participant, an underlying connection is generated and iterated by shared clinical ground; the outcome is a clinician-patient type bond that influences clinician-researcher suppositions and actions within the research relationship.

Throughout the results, clinician-researcher is usually abbreviated to researcher, and patient-participant to participant. In the first-person accounts, the patient-participants were typically participants in a larger research project and the researcher is reflecting on their clinician-researcher dual-role in the larger study. In all of the included empirical articles, clinician-researchers were participants in research about dual role; in addition, two of the empirical articles also included patient-participants ([18, 19]; see Table 1) but we only draw on findings from clinician-researchers who participated in those studies.

### Clinical patterns

#### Theme 1: *clinical queries*

If a participant asks a clinical question in a research setting, the researcher may interpret this as a straightforward request for information or reassurance. While researchers talk about clinical queries as an instance of dual-role, this experience does not usually cause much tension for them because they have the clinical knowledge to address this type of question or they can explain research boundaries and suggest an alternative resource for addressing this. Further, this dual-role experience is commonly expected and planned for, and researchers may feel confident their actions are within already agreed role boundaries. In addition, some researchers are comfortable addressing simple and reasonable questions, seeing this as an appropriate form of reciprocity.

*I had held a very fixed image of a nurse researcher as being someone who followed rigid rules of an imagined research persona. As I searched for answers to my role conflict*, *it became apparent that self-disclosure or intervention did not equate to high treason and that it need not invalidate the study. Thus, if participants asked me treatment-related questions I could offer answers… and provide details of agencies that might prove helpful.* [[3], p. 302]

Discomfort may arise if researchers want to respond to such queries but feel unable, believing their hands are tied due to such factors as fear of blurring roles or influencing data quality.

#### Theme 2: *perceived agenda*

Researchers may perceive that a question has another purpose than a simple request for information. Unlike *Clinical Queries* (perceived as a straightforward request for an educative or reassuring response) the researcher senses the question from the participant, or third party, contains an ‘agenda’.

##### Patient-participant agenda

Something in the participant’s statement or question (direct, indirect, or rhetorical) may lead the researcher to feel this is a request to agree with, or use their clinical expertise or influence to further, the participant’s agenda. For example, the researcher may be asked to acknowledge unmet needs, offer a second opinion, endorse a behaviour or expectation, act on behalf of the participant, or provide some other ‘advantage’.

Researchers commonly experience discomfort in response to perceived agendas, partly because they do not know for certain if their perception of a sub-text is true or mistaken. They may also feel torn between a clinical desire to respond to the participant’s appeal (perhaps feeling a need for reciprocity) and a concern that the response could create a false expectation of care, or there are competing loyalties to colleagues or service providers. At worst, this is experienced as an attempt to exploit the researcher, which is particularly distressing if there is a prior clinician-patient relationship. Hamberg and Johansson (1999), two family physicians who could certify people as medically unable to work, interviewed their own patients for a grounded theory study of women with unexplained musculoskeletal pain, and noted:

*Vera is challenging the rules for being certified for sick leave, and as physicians we had objections. In the margin of the transcript we had noted, “She can’t be sick-listed because she manages to work full-time! … How come she asks me such a bold question?”* [[20], p. 461]

##### Third party agendas

Others with a vested interest, such as the referring clinician or a family member, may directly or indirectly ask the researcher for research-generated information. For example, this may be an appeal to report back to a health professional about the participant’s health, convey information about the adequacy of care received, or reveal another person’s account of events. Tension occurs when research confidentiality and a desire to maintain collegial relationships are at odds. In addition, the researcher can feel conflict if declining to share information might jeopardize the research process.

*The difficulty is when they give you the names and then they say “Let me know if there are any problems”. Well that can be a bit difficult because by saying “No, unfortunately I’m not able to do that” then they might stop referring so many patients.* [[21], p. 426]

#### Theme 3: *helping hands*

Researchers may feel a strong sense that they are being asked or expected to use hands-on clinical skills to help participants or colleagues in the provision of patient care. There is a desire to help (whether acted on or not) for reasons such as: wishing to reciprocate, identifying with the clinical situation or need, feeling a professional duty of care to promote or advocate patient wellbeing, or being on the spot and having the clinical skills to help or ‘pitch in’.

*When call alarms are activated, and at times go unanswered, my natural inclination (or perhaps, conditioned response) is to go and answer them. I am finding it unsettling knowing that someone is needing assistance and yet staff are busy and cannot attend… This is becoming very frustrating when, with my ‘clinician hat’ on, I could easily be of assistance to both the woman and the staff member.* [[22], p. 56]

Despite informing others of differences between clinician and researcher roles, researchers may feel that others still do not understand the role differentiation and this is evidenced when others seek or expect the researcher’s help clinically. This is especially so when researchers are known to have expertise in clinical areas and in their health professional role would assess and respond to patient needs. Although researchers can pre-set limits on when it is acceptable to help, these may be challenged in unplanned situations with unmet patient needs. At times, the researcher feels an internal imperative to intervene clinically to prevent or manage a patient event.

*I was left alone in a room with six ventilated neonates. One of the neonates was very unstable and I was forced into the nurse’s role. I was ethically obligated to act when a neonate dramatically desalinated… [a life threatening situation]… When the nurses returned to the room they were quite happy with the fact I had had to intervene. However, since then, I make it quite clear that I am not legally covered to take on the nurse role.* [[23], p. 37]

Even so, not all agree that a researcher is obliged to act; regarding the above example, when the situation was presented to other clinician-researchers as a vignette, one respondent judged the researcher was not there as a nurse and “legally what she should have done was ring the buzzer three times and summoned whoever was supposed to be caring for the neonates” [[24], p. 66]

Researchers typically feel some conflict about helping at all, or their degree of involvement as participant observers in methodologies such as critical ethnography (e.g. Groenkjaer 2002 [25]) and grounded theory (e.g. Bonner and Tolhurst 2002 [26]) where researcher participation in clinical activity is deliberate. In giving assistance, researchers know they are acting as clinician in a research role, and question the professional, legal and methodological implications of their involvement. Conversely, not helping, despite having the clinical skills to do so, can leave researchers with lingering feelings of frustration and guilt, and concerns about whether they fulfilled their professional obligations to patient well-being.

#### Theme 4: *Research or therapy?*

Researchers are often concerned that participants have difficulty in distinguishing a clinician-patient relationship (clinical consultation) from the researcher-participant relationship (research contact). Reasons for disquiet are that the researcher suspects the participant experiences, or expects to experience, the research as therapeutically beneficial; researchers are also concerned that participants could experience harm from research. Thus, researchers wondered if participants agreed to take part in research because they expected to receive personal benefit (therapeutic misconception, see Appelbaum et al. 1982 [27]) or thought their future care would be harmed if they did not take part. Also, some participants appeared to debrief puzzling or distressing experiences with the researcher — the empathetic clinical expert — but researchers were not sure if this helped or unwittingly inflicted harm by generating a situation that required therapeutic intervention.

*I was never really sure how they really felt… sometimes they said it was the first time they’d been able to talk about it … but I’m not sure …I mean for some of them … the cancer was all behind them and then we come along and open it all up again … one or two were really quite upset by the experience … it really worries me.* [[21], p. 430]

Being known by the participant to be both clinician and researcher is sufficient in itself for researchers to experience dual-role and worry about therapeutic misconception. Potentially the participant may expect a clinical response or follow up that is not part of the research, and the power differential between clinician and patient may leave the participant less able to protect themselves from harm in the research setting. Although researchers did not seem to think participants’ expressions of intense or unexpected emotions was automatically accompanied by expectations of a therapeutic (counselling-type) response, the researcher often struggled with dual-role feelings; they usually experienced corresponding heightened emotion themselves and wanted to help the patient or themselves feel better. Many accounts reported researchers were left with a persistent “sense of unfinished business” [[28], p. 174] and a continuing concern about how the participant copes after the research is complete. Researchers were also concerned that the use of clinical skills (e.g. attentive listening and reflecting back) or their own emotional engagement impacted on the authenticity of the data or interpretation.

*I experienced a conflict of emotions as to how I should act. I realised the significance of Ann’s experiences and feelings, but at the same time I was in the position of power and I was aware of the vulnerability of Ann in this situation. I decided to turn off the tape recorder - - - I may have acted in haste and denied Ann the opportunity for her voice to be heard. In my mind I was juggling the research interests with the responsibilities of being a researcher, a midwife, and an empathetic human being.* [[29], p. 654]

Other researchers felt they could blend the roles; “I wondered if what I was doing as a researcher was any different to my usual role of listening empathically and trying to make meaning out of what was being said … I decided that research interviews were therapeutic and allowed the participants to tell their stories” [[23], p. 37] Again, when this example was presented as a vignette in a later study, it was clear not all clinician-researchers would agree: “She’s lost the plot. - - - I think she’s really not seeing what research is all about in this type of setting. I don’t think you can do it and be totally objective” [[24], p. 66]. Nevertheless, some researchers have a different perspective on research as therapy. For example, some theoretical positions and accompanying methodologies (e.g. feminist) are emancipatory in which the research deliberately seeks to offer opportunity for patient-participants to benefit from the research by conferring legitimacy to experiences, empowering participants, and facilitating reciprocity (e.g. Hamberg and Johansson 1999 [20]).

#### Theme 5: *the uninvited clinical expert*

Researchers often find themselves in situations in which they have ‘automatically’ used their clinical knowledge or reasoning and, as a consequence, identify concerns about participants or study protocols.

##### Incidental clinical findings in research

Through dialogue, observation, clinical notes or other ways of collecting data, the researcher’s instinctive and unintentional use of clinical reasoning may raise concern about the participant or their care. That is, dual-role arises because information gained in the research setting has clinical utility or implications. For example, the researcher may identify a risk or unmet health need, misunderstanding or misuse of some aspect of care, or inadequacies in care delivery.

*If a participant told us that her low back pain had changed lately, we would immediately think of new medical investigations* [[20], p. 460].

Unlike *Perceived Agenda,* the participant does not deliberately express concern and may be unaware of the issue. A troubling aspect for the researcher is the uninvited nature of the discovery. Moreover, in cases of substandard care, they may feel shame on behalf of their profession or colleagues. Unlike *Clinical Queries,* in which a participant makes a direct and straightforward request for help, these unsolicited needs do not have the same sense of preparedness and/or legitimate action for the researcher.

*Was I a spy? What should I have told the nursing home managers about what I saw? - - - What should I do when I observed a staff member being short-tempered with a resident, or failing to provide professional care?* [[30], p. 45]

When an incidental clinical issue was identified, feelings of dual-role seemed greater when the researcher had to decide whether to act on this knowledge or not; not acting can cause the researcher to question whether they did the right thing, similar to reactions reported under *Helping Hands*.

##### Research-related decisions

Sometimes, researchers are aware of dual-role when they have used therapeutic criteria and clinical expertise to make research-related decisions on behalf of a patient. This would be labelled therapeutic misdirection [17] because the researcher has misdirected the research process in an attempt to provide therapeutic benefit for the patient (distinct from participants having therapeutic misconception, as reported under *Research or Therapy*). For example, the researcher may decide not to offer study information to an eligible potential participant because they believe it is not in the person’s best interests to take part, or disregard minor entry criteria or make low-risk adaptations to the research protocol believing that a patient will benefit from research participation. This potentially biases the research sample if population representativeness is being sought.

*It’s usually a judgmental issue which in your own clinical experience you may feel is detrimental to the care of the patient or in some case; it may be because you think something is not warranted to be done that is dictated by the protocol - - - and so you may question whether doing those investigations is required.* [[7], p. 5]

Therapeutic misdirection is the researcher’s internal dialogue by which clinical expertise is applied to research criteria for the patient-participant’s clinical benefit. For some researchers, taking responsibility for judgments about the likely clinical benefit of the research to a patient-participant is construed as a research role, while others distinguish research from clinical decision-making. Tension seems inevitable here – a researcher with relevant clinical expertise has the skill-set for implementing a clinical research protocol, and adherence to research ethics and methodological demands may be at odds with their clinical opinion about a patient-participant.

*For me, the issue is always what I think is best for the patient and so, you know, if something has to give, it is the protocol.* [[7], p. 4]

Other, less common, instances of reported dual-role include the researcher recognising that information obtained during the therapeutic relationship is useful as study data, and awareness of problems with the research protocol due to their clinical expertise. For example, the researcher notices important clinical variations in the delivery of an intervention that could undermine data quality or interpretation. In the latter case, the researcher’s professional loyalties and personal experience of clinical realities create tension about reporting or acting on these protocol deviations.

### Connection

As an over-arching theme, Connection captures the clinician-patient type bond that manifests within the primary researcher-participant relationship; the catalyst for this is that both researcher and participant have experience of the clinical context. The clinical patterns outlined above may potentiate a clinician-patient type connection.

#### Theme 6: *clinical assumptions*

Researchers and participants typically both have an experience of clinical care for the health issue in question. The extent of common ground varies substantially; where there was a prior clinician-patient relationship there are known patterns of interacting in a specific clinical setting, and where there is no history, common ground can stem from familiarity with health conditions, interventions or professions. Common ground creates opportunities for assumptions of shared understanding during the research process, which may be genuine or presumed.

*The person who was once a patient, or may still be a patient in a different healthcare context, holds memory of the nurse-patient relationship and is often willing, if the situation arises, to grant a researcher who is a nurse a privileged relationship; an instant familiarity that results from a shared understanding of health, illness and the body.* [[6], p. 156]

Participants appeared to make assumptions about the researcher’s clinical expertise and could act in ways seemed to presume the same boundaries of confidentiality or intimacy in research that typify clinical consultations. The researcher may be uncomfortable that the participant apparently assumes their clinical expertise in the research topic is greater than is the case and be “concerned that I would be seen as being more capable than I felt” [[30], p. 44]

Assuming some knowledge in the other is probably reasonable to avoid lengthy explanations or questions that both parties find unnecessary, irritating, or time-consuming.

*As participants indicated their discomfort with this different approach, at times the nurse felt they had to work hard to keep then engaged in the process: …I did find sometimes … it was like trying to teach them to suck eggs - - - they’d look at me as if to say “Are you honestly asking me this?”* [[31], p. 594]

Seemingly, it was after a research contact that researchers were mostly likely to be bothered by potentially inaccurate or unsubstantiated assumptions, unquestioned preconceptions or premature interpretation. The stimulus was commonly the data analysis phase, when the researcher realized their assumptions and interpretation of the data may not be clear to others.

#### Theme 7: *suspicion and holding back*

The researcher may suspect that the participant is holding something back; this is sometimes overtly stated by the participant but usually inferred by the researcher from a participant’s guarded speech or behaviour. The participant may seem suspicious of the underlying purpose in the research or what use will be made of the information, afraid of failing to meet the researcher’s expectations, or concerned that what they (the participant) say or do (or fail to say or do) has consequences for future clinical care. This sense of distrust can provoke anxiety in the researcher when: (a) it is interpreted as a signal that the participant felt coerced to take part, or (b) the participant’s holding back affects the quality of data.

Conversely, the researcher may be reassured by criticism of services, people, or care because this suggests that the participant is not holding back. However, as a clinician such disparagement can be uncomfortable to hear.

*I was relieved that some of them felt able to criticize the services of the hospice. In this respect, I made a particular effort to remain neutral. I resisted all instincts to defend the hospice or any other health and social services - - - or indeed to confirmed their criticisms. I was left, however, with feelings of discomfort and disappointment.* [[32], p. 35]

#### Theme 8: *revelations*

If a participant reveals intimate information (more than was expected or sought) that has important clinical implications, the researcher can feel that a response is needed and concern about how best to respond without confusing researcher and clinician roles. The revelation, which may well be accompanied by heightened participant emotion, may evoke a corresponding heightened emotion in the researcher and “this unintended expression of empathy seemed to encourage them to greater levels of disclosure”. [[28], p. 174]

*One or two residents told me of experiences of treatment that I had hoped never to hear. Their stories were given in confidence, and on condition that I did not intervene in any way. I respected their wishes, but didn’t I have some role as a fellow human being here, let alone as a health professional?* [[30], p.45-6]

At the extreme is the confessional utterance in which the participant reveals information that elicits in the researcher a powerful sense of clinical, ethical, or legal obligation to act, such as suicidal ideation or likely elder abuse by a family member. Revoking confidentiality when obliged by duty of care incites considerable angst for the researcher.

*I became distressed. I felt the participant needed to have immediate psychological assistance and that he had trusted me, almost appealing to me to help him - - - I decided to tell the nurse in the centre that in my professional opinion I felt this person should see a psychologist immediately… I left wondering what was the eventual outcome for this person, I still reflect my feelings of despair.* [[23], p. 37]

#### Theme 9: *over-identification*

Two types of over-identification may occur – within, and with other. That is, the researcher can over-identify with their clinical self, or the researcher can over-identify with the participant.

##### Over-identification with the patient-participant

A researcher may become intensely engaged with a participant, and common ground becomes boundary permeability with transference or projection of researcher feelings. For example, when the participant is distressed the researcher feels distress, and the researcher may become protective of the participant, feeling the participant is dependent on him or her. One consequence is that researchers can become anxious about how to disengage their supportive presence from participants at research completion. Rather than leave the field with the feeling of unfinished business, the researcher may extend the timeframe and boundaries of the relationship, remaining in touch with participants for some time. In particular, participant observation (e.g. Patterson 1994 [33]) and longitudinal data collection (e.g. Sterling and Peterson 2005 [34]) may encourage boundary permeability between researcher and participant. Considerable effort may go into avoiding this form of over-identification.

*I did not know if I caused distress when these areas were discussed. For some individuals the implications of their impairments did not appear to cause distress when discussed, and therefore I felt that I must be wary of projecting my own feelings onto them - - - No one could help feeling a great deal of compassion in this situation, but it was again important to convey the responses and experiences of the respondents, and identify and acknowledge my own for what they were.* [[35], p. 189]

##### Over-identification with the clinical self

The researcher may over-identify with their sense of clinical self, or the clinical environment within which they are carrying out research. That is, the researcher can feel too close to have an ‘outsider’ perspective in the research and be ‘blind’ to the phenomenon or setting being studied.

*She might have observed with her “nursing glasses” instead of “researching glasses,” a situation that might have caused cultural blindness. This was probably the case at first, as it was difficult for her to develop any kind of distance from a situation she knew so well. She dressed like a nurse, and as she assisted the nurses in their work, she might at times have paid more attention to patient care than to research.* [[36], p. 700]

Over-identification with the clinical self can affect data collection; for example, experienced nurses finding “it difficult to limit their enquiry to a structured format” required by a research protocol [[37], p. 190] or feeling “embarrassed and compromised by this story, worrying that Nede’s GP’s behaviour may have reflected on her profession, generating a reluctance to hear more” [1], p. 4]. Data analysis may be constrained, such as feeling compelled to massage or omit findings that betray or cast a negative light on the researcher’s own profession or colleagues, especially when reporting back to clinical colleagues.

*I am beginning to feel like it is a breach of trust to report any negatives about the way midwives conduct their work. I feel that the midwives have generously offered to let me observe their practice and it subsequently feels wrong to highlight the areas of poor practice observed.* [[22], p. 56]

Realisation about over-identification tends to occur when looking back reflexively and researchers can feel they lacked insight about what was happening at the time. The main concern is for trustworthiness of study findings.

*Was I analysing participant narrative through the eyes of a researcher or through the eyes of a nurse with a different knowledge base of the healthcare system? I was now aware that my professional socialisation could be getting in the way. I went back to the original data and found that although I was analysing the data from the study participants, I was also slanting them from my perspective as a nurse - - - I realised I was being more critical of the service experienced by participants than the participants were themselves.* [[3], p. 298–99]

#### Theme 10: *manipulation*

A researcher may intentionally engage in behaviours that create or foster a participant’s or colleague’s experience of clinical trust in order to advantage the research. In the most extreme case, this may involve the researcher deliberately abusing a clinical trust relationship through deception or exertion of power, with potential for manipulation and coercion. An example of concerning behaviour may involve the researcher wearing a uniform (as in the previous theme) to increase the likelihood of the participant consenting to participate in the research, or talking about information ordinarily confined to clinician-patient relationships. Another, is feigning relationship to increase participant willingness to provide data that they might otherwise withhold.

*Although some carers may have revealed more than they had anticipated about their experience and emotions, I do not think that I manipulated them into disclosure by being too intimate or faking friendship.* [[32], p. 34]

Potential for manipulation was recognised in relation to power imbalance, which was possibly greater when the researcher was also a clinician.

*Many of the working-class respondents were deferential: the title ‘Doctor’ was often used and I was introduced by several interviewees to family members as ‘the doctor’. One respondent apologized for taking up my time, even though the interview took place at my request* [[38], p. 73]

Researchers seemed to persistently question the motives underpinning their behaviour and whether they were exploiting the participant or situation to their advantage. In particular, some methodologies, such as emancipatory or participatory approaches, specifically encourage researchers to grapple with power relationships and reciprocity. For example, Gardner (1996) offered two interview excerpts contrasting clearly demarcated clinician and researcher roles with another where she “was more conscious of myself as a nurse researcher and so the transition to intimacy and reciprocation was seamless” [[6], p. 157] Thus, on one hand, there is concern about the abuse of power. On the other, there may be intentional embracing of dual-role in order to use the privileged position to advocate for and empower participants, as well as elicit rich data.

## Discussion

Our review process, and resulting typology, identified two main catalysts for dual-role experiences: clinical patterns and connection. No judgment of clinician-researchers’ thoughts, feelings, actions, or reflections is intended by our review. Rather, the purpose of the typology is to reliably capture the main ways that dual-role is experienced by clinician-researchers, creating a focus for continuing discussion about how to manage the implications of dual-role.

Our central finding, having reviewed the systematically retrieved reports of clinician-researcher experience of dual-role, is that clinician-researchers cannot adopt a wholly non-clinical research identity. Indeed, their clinical expertise may be the very reason they are recruited to research roles [1], or initiate research to investigate clinical questions [5]. In fact, Gardner (1996) has argued for the advantages of deliberately positioning ones-self as a clinician-researcher to enable patient-participants to “talk with freedom and comfort” giving data that are “full, rich, and thickly described” [[6], p.157].

The root cause of dual-role is the interaction of the researcher’s clinical socialisation, orientation and experience (knowledge, skills, ethical and professional obligations) with the participant’s prior experience of the patient role (with associated expectations). Consciously and subconsciously, the clinician and patient blueprints are brought into the research encounter, creating shared ground and a connection that resembles a clinician-patient relationship.

Clinician-researchers may find it artificial and insincere to attempt to dissociate from their clinical identity, and have concerns about authenticity (both for their relationship with participants, and for the study) (e.g. Clancy 2007 [39]), while realising holding ‘both’ identities fosters feelings of dual-role. Wearing ‘glasses’ is a common metaphor illustrating the difficulties of role separation, such as the previously cited analogy of “nursing glasses” and “researching glasses” [[36], p. 700]. ‘Hats’ are another allegory; “one hat is the hat I put on when I’m your doctor to take care of you. And the other hat is the hat I put on when I’m trying to see if something new works, or to find out how to give it….and when I’m taking care of you I wear both hats” [[18], p. 705]. This quote from Easter et al. (2006) clearly shows the inadequacy of common metaphors such as glasses or hats, both of which can be shed at will. Our central finding of the inability to fully separate the researcher and clinician roles suggests other metaphors such as ‘clinical eyes’ or ‘clinical skin’ provide a better sense of the deep-rooted and embodied clinical identity that cannot be shed in a research context.

Others also blur the boundary between clinician and researcher; dual-role is produced by external as well as internal influences [2]. External influences include the expectations of participants and others, such as clinical colleagues and participants’ families. Holloway and Wheeler (1995) suggest that patient-participants may not understand the duality and dichotomy of the clinician-researcher and patient-participant roles and will expect empathetic clinical care from a professional who also has the title of researcher [4]. The socially constructed identities of clinician and patient means that participants bring their own preconceived notions about the clinician-researcher role, independent of the desired positionality [22]. These preconceptions mean that patient-participants may confuse the distinction between research and treatment goals, and take part in studies in order to gain clinical benefit (therapeutic misconception) [27]. Even after carefully explaining role differentiation, researchers may feel that patients still find it difficult to distinguish their clinical and research roles [40]. Role confusion is not limited to patient-participants. Sterling and Peterson (2005) state that “although families accepted the scientific and investigational nature of the study, in their eyes the researchers were seen first as nurses” [[34], p. 48]. Misunderstanding also extends to clinical colleagues, with situations in which the clinician-researcher is expected to help out clinically while present in a research role.

Within research, the clinician-researcher’s internal boundary blurring essentially arises from the clash between the clinical mandate to act in the individual patient-participant’s best interests (beneficence) while not causing harm (non-maleficence), with the scientific mandate to pursue knowledge with appropriate rigour [2, 41]. Researchers have an obligation to protect the interests and welfare of research participants, as spelled out in the Declaration of Helsinki [11]. Clinician-researchers are familiar with the notion of non-maleficence from the clinical arena and can apply this to research settings [42]. However, while some values such as protection from harm applies in both the research and clinical area, it should not be assumed that all values apply equally in both areas [43].

Attempts have been made in the bioethics literature to establish descriptions and models for the relationship between clinical and research roles; the two dominating theoretical models are the Similarity and Difference positions [7]. The Similarity position posits research as a subsidiary element of health care, and the research therefore takes place within the professional obligations and norms of clinical practice. The Difference position contends that the aims of clinical care and research are different and thus the prevailing ethics of the two activities are also different; the two positions must be separated to prevent therapeutic misconception in the minds of clinician-researchers and patient-participants [7, 18]. Czoli et al. (2011) found in interviews with 30 paediatric physician-researchers in Canada that strict adherence to the theoretical positions of Similarity or Difference is not in accord with the ways that clinician-researchers described their lived experiences of dual-role [7]; the models give weight to one set of obligations at the expense of the other whereas dual-role means devising strategies and ways of thinking that balance both. The clinician-researchers did not talk about strict Similarity or Difference positions, “perhaps indicating that a complete divorce between the two practices is uncomfortable for or undesired by physician-researchers” [[7], p. 5] Accordingly, they argued for a middle ground position which recognizes a fundamental difference between clinical and research norms and incorporates the similarity position of preference for clinical norms. Congruent with our interpretation of data in this review, this middle ground approach better reflects what can be reasonably expected of clinician-researchers in real-world practice, and at the same time maintains the expectations of high ethical conduct. In effect, dual-role might best be understood as a coherent moral identity that recognizes both sets of obligations, rather than oscillating between the two roles [7].

In pursuing and developing guidance for high levels of ethical conduct, some occasions for dual-role have received particular research and scholarly attention, such as the process of recruitment and informed consent [5, 44]. While some instances of dual-role may be more evident in one paradigm — such as the overlap between data collection procedures and clinical procedures in quantitative research, and the potential for role confusion arising from the degree of interaction between clinician-researchers and patient-participants in qualitative research — the potential for ethical issues consequent on dual-role arise from the whole typology in both paradigms. For example, boundary issues arising from close engagement between clinician-researcher and patient-participant are postulated to inundate qualitative research with ethical issues not found in quantitative research [45]. An alternative, plausible, explanation is that qualitative studies are no more fraught with dual-role than quantitative studies; rather, researchers may be particularly alert or sensitised to dual-role through methodological demands for attention to positionality and reflexivity in qualitative research. Our review found, predominantly, first person accounts of dual-role arising in qualitative studies (congruent with expectations of researcher reflexivity) and a few primary studies that included clinician-researchers involved in quantitative research. Analysis of the data revealed similar dual-role tensions in both paradigms, suggesting clinician-researchers need to consider each component of the typology regardless of research type.

The emphasis, in research protocols and ethics applications, on accepted (and ethically justified) means of separating clinical and research roles during recruitment and consent processes is insufficient. The typology demonstrates that many experiences of dual-role occur during data collection, and some are unexpected. Research preparation and monitoring needs to encourage clinician-researchers to grapple with the wide range of ways in which dual-role shows up, whenever it occurs. However, while ethics committees look for evidence that researchers understand the principles of ethical research, they may not drill down to the fine detail of all anticipated instances of dual-role [2, 46] and cannot predict the unexpected. Madjar and Higgins (1995) used an analogy by bioethicist Albert Jonsen to illustrate this distance between ethics committee requirements and the realities of clinical research: balloons and bicycles [46]. The balloonist (ethics committee) sees the wider landscape and understands the theory and principles of the bigger picture, whereas the cyclist (researcher) experiences the reality of bumpy roads, potholes and obstacles to applying the theory and principles. Ethical decision-making is not a simple matter of slavishly following guidelines, but rather acknowledging variable factors involved [45]. While ethics committees and research protocols serve to provide protection, in the end each clinician-researcher requires a degree of ethical reflexivity to ensure they are ethically aware of and sensitive to the issues when acting in a dual role [5].

If the inevitability of dual-role is accepted, and dual-role is considered to have benefits as well as challenges [47], then planning for it and having review processes in place to reflect on pros and cons may assist with managing it well both ethically and methodologically. Even so, there is probably no way to reliably anticipate all specific events that will trigger dual-role experiences because so much depends on the research topic and setting, the methodology and design, the individuals, and the unexpectedness of research. For novice researchers, there are benefits in drawing upon the experience of others prior to entering the research field [8]. However, whilst concrete examples of dual-role experienced by other clinician-researchers may be helpful, the typology prompts consideration of a broader range of stimuli for dual-role than any one researcher may have encountered.

This review, and typology, represents a move toward understanding how the challenges of dual-role are evidenced for clinician-researchers. This expands upon the larger body of literature which has tended to focus on why specific instances of dual-role are experienced (e.g. Allmark et al. 2009 [44], Edwards and Chalmers 2002 [5]). Representing the real-life experience of dual-role, the typology does not eliminate tensions between patient care and scientific rigour. Rather, the typology recognises and encourages awareness of the often messy personal experiences of dual-role that are starkly contrasted with the abstract nature of theoretical models and sanitized reporting of most research. The intent of the typology is to promote awareness and responsibility for ‘what is’ — both the potential benefits and challenges of dual-role — so that all those involved in the research process have confidence in the integrity, understanding, and self-awareness of clinician-researchers to produce ethically and methodologically sound research [7].

We suggest the best use of our typology is as a discussion framework when designing and implementing research. The typology offers an opportunity to focus on the education and training of clinician-researchers in ways that “deal directly with concrete situations in which the dual commitments to research and clinical care might conflict and how clinicians in the research context should respond to such tension” [[41], p. 6]. Research team meetings and other forms of supervision (for research students, and novice researchers) are an opportunity for debriefing and guidance [48]. Discussion with others from different backgrounds may help uncover alternative and previously unconsidered perspectives [47]. Supervision can help identify and explore ethical and practical dilemmas that occur due to blurred roles [49]. Importantly, regular review also attends to the needs of the clinician-researcher, and ensures a process to explore and manage any boundary blurring that occurs, and “ensure that researchers [our emphasis] are not adversely affected by their participation in research” [[50], p. 867]. In sum, discussion and increased awareness of dual-role may not only avoid difficulties arising from it but actively enhance the research process and outcomes.

In Table 2, 10 questions offer starting points for discussion to raise awareness of the common ways in which dual-role shows up and we also pose one further question about post-research clinician-patient relationship; we found no examples of this in the analysis. Yet, there are dual-role implications if researcher and participant meet again as clinician and patient, such as clinical use of privileged and confidential information gained in the research setting. Haigh et al. (2005) noted, although gave no specific example of it, that “dual roles did not end with the discharge of the patient from hospital, but existed for as long as contact with the RA remained” [[19], p. 79]. We encourage clinician-researchers to consider future risks to themselves and patient-participants (Table 2).

**Table 2:** Questions for clinician-researchers planning research with patient-participants

Using your clinical skills
1. When is it appropriate to address a clinical question from a patient-participant? 2. What will you do if you think the patient-participant or another person (such as their carer) is asking you to use your clinical influence or expertise? 3. When you feel the urge to give physical assistance, what makes it appropriate or not? 4. If you, incidentally, identify a clinical issue or patient-participant need, how will you manage this? 5. When is it acceptable for a patient-participant to receive personal (therapeutic) benefit from taking part in research?
Creating a relationship with the patient-participant
6. What assumptions can you make about shared understanding based on shared clinical ground? 7. What risk is there of using a trust relationship for your own ends? 8. What signs are there that a patient-participant might feel coerced or obliged? 9. What will you do if a patient-participant reveals intimate information of concern? 10. How will you know if you are ‘too close’ to see?
After the research
11. What happens if you and the patient-participant meet again, this time as clinician and patient?

A limitation of this review is the literature search may not have found all first person accounts or primary investigations of dual-role experiences because database indexing was heterogeneous and there were no common keywords in use to denote the concept. However, our combination of scoping searches and search terms tailored to a range of databases along with reference list checking provided a relatively diverse body of clinician-researcher literature to review. Data saturation is not determined by volume of text or details of individual events, but rather by saturation of characteristics within categories, with a level of interpretation that allows one to make sense of a complex phenomenon [51]. The dual-role phenomenon as experienced by clinician-researchers was captured cohesively and consistently from the included studies, from those involved in quantitative and qualitative research, and research from multiple disciplines and fields although qualitative nursing literature predominated. Based on our presentations and discussion of the typology with bioethicists and clinician-researchers, the constituent themes are unsurprising and recognisable. Future research with clinician-researchers on the themes within our typology could be applied to fields and aspects of clinical practice that are less represented within the existing literature that is currently dominated by nursing research. A strength of the existing literature is the variation of methodologies from which the dual-role experience is reported, and continued attention to diverse methodologies would benefit future research.

Clearly, experiences of dual-role may not be confined to clinician-researchers. For example, non-clinician researchers investigating sensitive topics may experience conflict between what they feel are the demands or expectations of robust and ethical research versus their moral obligations in relating to others [50]. However, it is the involvement of patients in the research that is the primary catalyst for triggering the overt appearance of clinical identity within the research role, since clinician-researchers have a heightened sensitivity, conditioned response, and duty of care to prioritise the needs of patients. There are opportunities to test and expand the typology in other areas, such as clinician-researchers investigating other clinicians (research on peers that does not involved direct patient-participant contact or observation), dual-role experiences in research with vulnerable populations or on sensitive topics, and dual-role from the perspective of the patient-participant.

## Conclusion

Once a clinician, always a clinician. Clinician-researchers cannot shed their clinical skin, and acknowledging the inevitability of dual-role is important for them, their supervisors, and ethics committees. Clinician-researchers use their clinical skills in research in ways that create shared clinical ground with patient-participants, and this sets up a secondary relationship that resembles that of clinician and patient. Clinician-researchers’ ingrained orientation to patients’ needs is often experienced in tension with their research role, especially research ethics and methodological demands. The potential for dual-role to raise ethical and practical issues needs to be carefully considered, and skilled supervision is essential for novice researchers. Using the typology to discuss the ways in which dual-role is typically experienced could encourage clinician-researchers to: grapple with the unavoidability and implications of dual-role; recognize and hold and review both sets of obligations throughout and after the research process; and ensure that clinical and research roles are not artificially separated in research protocols, ethics applications, and research practice.
